# Mixed methods analysis of an interdisciplinary intervention to promote balance confidence in lower limb prosthesis users

**DOI:** 10.3389/fresc.2025.1626051

**Published:** 2025-09-01

**Authors:** Noah J. Rosenblatt, Kristin L. Schneider, Steven A. Miller, Kavork Hagopian, Sarah Hagg, Christopher Reddin, Rachel Churchill, Gregory M. Dams, John E. Calamari, Aaron Stachowiak, Matthew J. Major

**Affiliations:** ^1^ Dr. William M. Scholl College of Podiatric Medicine’s Center for Lower Extremity Ambulatory Research (CLEAR), Rosalind Franklin University of Medicine and Science, North Chicago, IL, United States; ^2^ Psychology Department, Rosalind Franklin University of Medicine and Science, North Chicago, IL, United States; ^3^ Department of Physical Therapy, Rosalind Franklin University of Medicine and Science, North Chicago, IL, United States; ^4^ Department of Physical Medicine and Rehabilitation, Captain James A. Lovell Federal Health Care Center, North Chicago, IL, United States; ^5^ Doctor of Nursing Practice: Psychiatric Mental Health Nurse Practitioner Program, Rosalind Franklin University of Medicine and Science, North Chicago, IL, United States; ^6^ Department of Physical Medicine and Rehabilitation, Northwestern University, Chicago, IL, United States; ^7^ Department of Biomedical Engineering, Northwestern University, Evanston, IL, United States; ^8^ Department of Mechanical Engineering, Northwestern University, Evanston, IL, United States; ^9^ Department of Physical Medicine and Rehabilitation, Jesse Brown VA Medical Center, Chicago, IL, United States

**Keywords:** fear of falling, physical therapy, cognitive behavioral therapy, balance, function, quality of life, amputation, community participation

## Abstract

**Introduction:**

Low balance confidence, i.e., low self-perception in ones' ability to maintain balance while performing activities, is prevalent among lower limb prostheses users (LLPUs) and can affect community participation and quality of life (QoL). Although low balance confidence can manifest from poor function, it also depends on one's beliefs in their abilities to engage in activities, which need not reflect actual abilities. Increasing low balance confidence and associated participation limitations requires approaches that address its' physical and psychological underpinnings.

**Methods:**

A randomized controlled trial was conducted to evaluate the initial effectiveness of a multicomponent intervention to target balance confidence in LLPU. Nineteen adults with ≥6-months experience using a prosthesis for unilateral, transtibial amputation, and with low balance confidence (Activities-specific Balance Confidence (ABC) scale scores ≤ 80) completed up to eight intervention sessions following an established protocol, which integrated physical therapy exercises (primarily virtual reality active gaming) and cognitive behavioral therapy strategies, or eight weeks of at home-seated exercises. Outcome measures, collected before randomization, and 0- and 16- weeks after completing the intervention/at-home exercises, addressed four domains: (i) balance confidence—the ABC scale, modified Gait Self Efficacy scale and the Fear of Falling Avoidance Behavior Questionnaire; (ii) community participation—sections of the 36-Item Short Form Survey, sections of the Community Reintegration of Injured Servicemembers scale, the Frenchay Activity Index and step counts; (iii) QoL—the wellbeing scale of the Prosthetic Evaluation Questionnaire; and (iv) function—the Berge Balance Sale and the L-Test of walking. Statistical tests compared baseline and post-training assessment scores between groups, and individual responsiveness was evaluated by comparing change scores to minimum detectable change (MDC).

**Results:**

Overall, results support the initial efficacy of the intervention, with at least one outcome in 3-of-4 domains (balance confidence, community participation and functional mobility) showing strong, significant group-level effects, or individual-level effects (>30% of participants having changes > MDC). Moreover, semi-structured exit interviews suggest participants perceived benefit from the intervention.

**Discussion:**

Integrating physical therapy exercises with cognitive behavioral therapy strategies to simultaneously address physical underpinnings and maladaptive cognitions around low balance confidence can meaningfully improve balance and walking confidence, as well as community participation. To the best of our knowledge the current study is the first to evaluate an intervention to specifically target balance confidence in LLPUs.

**Clinical Trial Registration:**

clinicaltrials.gov, identifier NCT03411148.

## Introduction

Low balance confidence, defined as low self-perception in ones' ability to maintain balance while performing specific activities, is a prevalent issue among lower limb prostheses users (LLPUs) that can affect community participation and quality of life (QoL) (1). In a cohort of 435 community-dwelling individuals who had been living with lower limb amputation for at least six months, 65% reported levels of balance confidence below the threshold at which intervention is advocated for non-disabled adults (2, 3). In the same cohort, low balance confidence was a predictor of participation in social activities, even after accounting for mobility capability and other prosthesis-related characteristics (4). Low social participation due to low balance confidence can negatively impact QoL (5, 6). Low balance confidence is also a strong predictor of whether a LLPU can attain a level of walking consistent with community-ambulator status and may be a precursor to increased disability (7). A review on older adults without amputation suggested that “Interventions to maintain appropriate levels of balance confidence must … be viewed as preventative … on the pathway leading to disability” (8). Nonetheless, “balance confidence is not directly addressed during prosthetic rehabilitation” (9).

Although it is logical to assume that low balance confidence manifests from poor functional abilities, this is not always the case. Balance confidence is a measure of self-efficacy and therefore depends on one's beliefs in their abilities to engage in activities, which need not reflect their true abilities. In fact, in LLPUs, measures of balance confidence and performance-based measures of balance are only moderately correlated (10). In addition, while walking ability in persons with lower limb amputation continues to improve following discharge from rehabilitation, balance confidence does not, suggesting the two may be independent (9). While treatment that focuses on improving walking ability is important, it may be insufficient to address low balance confidence and may fail to facilitate meaningful improvements in community engagement and QoL.

Interventions targeting low balance confidence and associated limitations in community participation may require approaches that simultaneously address both the physical and behavioral underpinnings of low balance confidence. A review of eight intervention studies in older adults that included balance confidence as a primary outcome reported more robust evidence supporting the benefits of multifactorial interventions, i.e., those which included a physical and behavioral component, compared to single-component interventions (8). A more recent review evaluated multicomponent interventions that specifically included cognitive behavioral therapy (CBT)-based strategies in conjunction with prescribed exercise/rehabilitation in non-disabled older adults. The authors reported significant, immediate effects of these interventions on fear of falling (a construct closely associated with balance confidence and often quantified using both balance and falls self-efficacy scales) (11).

Despite the potential for multicomponent interventions to improve balance confidence and related constructs in non-disabled older adults, such interventions have received limited attention in LLPUs. There is at least one published report regarding development of a multicomponent intervention to target balance confidence and increase community integration in LLPUs (12). This intervention consists of eight sessions that integrate physical therapy (PT) exercises, primarily virtual reality active gaming, with CBT strategies that addressed participant-specific avoidance behaviors and maladaptive cognitions related to low balance confidence**.** A previous case study demonstrated initial feasibility of the cognitive behavioral and physical therapy intervention (CBPT intervention), reporting that balance confidence, as assessed by the Activities-specific Balance Confidence (ABC) scale, more than doubled from the start of the intervention to a one-month post-intervention follow-up (13). Building off this work, the purpose of this study was to conduct a randomized controlled trial to evaluate the initial effects of this CBPT intervention on balance confidence, community participation, function, and QoL**.** It was hypothesized that: H1) LLPUs receiving CBPT would demonstrate greater improvements over time in balance confidence compared to those in the control (at-home exercises only) group; H2) LLPUs receiving CBPT would demonstrate greater improvements in community participation over time, due to reduction in balance-confidence-related barriers, compared to those in the control group; H3) LLPUs receiving CBPT would demonstrate greater improvements in balance and functional mobility compared to those in the control group; and H4) that LLPUs receiving CBPT would demonstrate greater improvements in QoL over time compared to those in the control group. Key informant interviews and thematic analysis were used to explore participant's experience with the CBPT intervention.

## Methods

### Participants

Participants were included in the study if they: were >18 years of age, had a unilateral transtibial amputation, had at least 6 months experience with using a prosthetic device, had an ABC score ≤80 (low balance confidence), and affirmatively responded to: “Do you have balance concerns that prevent you from engaging in activities that you would otherwise like to do?”. Subjects were excluded for: neurodegenerative diseases, currently being in PT for any reason, and inability to independently stand for 2 min (occasional light touch of an assistive walker was permitted). The last criteria ensured that the participant would be able to perform the PT exercises without an assistive device, which would interfere with the equipment being used.

### Recruitment

Participants were recruited using several methods. Local amputee support groups and veteran groups were sent information about the study and study team members visited groups to provide short recruitment presentations. We also searched medical records from four regional Veterans Affairs Hospitals for patients with unilateral transtibial amputation who used prosthesis and mailed them form letters indicating they may qualify for a study; mailers also included initial screening material (ABC scale and balance-concern question) that were to be returned if the person was interested in receiving additional information. We followed up with those individuals whose returned screeners that met inclusion criteria. The study was also posted on ClinicalTrials.gov.

### Screening

After providing written informed consent for this IRB-approved study, a certified prosthetist (CP) evaluated the LLPU to ensure proper prosthetic fit, well-functioning components, and a healthy residual limb. The CP recorded information about the individual's prosthetic history and current componentry. A study physician then performed basic physical screening to ensure it was safe for the participant to engage in the low-to-moderate levels of exercise dictated by the study protocol. A mini mental status exam was then performed to ensure a score of ≥25 (out of 30) (14).

**Figure 1:**
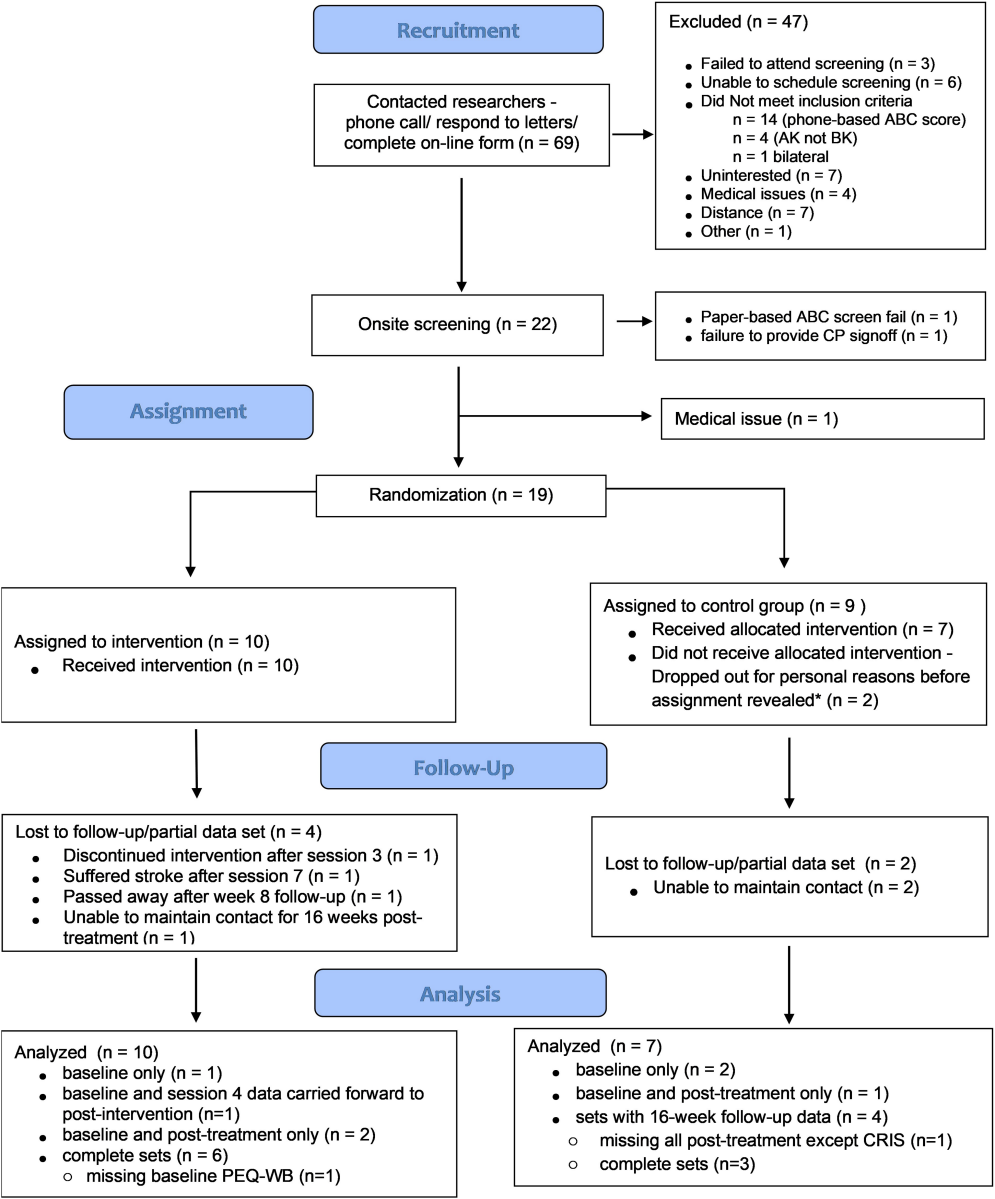
Participant flow chart. * Participants were assigned to a group and study team was informed of the assignment before it was disclosed to the participant.

It was not always possible to coordinate the availability of the participant with that of the study CP and the study physician. In the case where only one screening clinician could attend the session, a sign-off form was sent to the participant's CP or physician. If the form was not returned in a timely manner, attempts were made to facilitate an onsite screening session with the outstanding clinician. In one case, a recruited participant who received sign off from the study physician was never randomized to a group due to failure to obtain sign off from a CP (see Figure 1).

### Baseline assessments

After passing an initial screening all participants completed the ABC scale, the primary outcome for the study (see Table A1 in Supplementary Material for details on assessment tool scoring and psychometric properties). The scale was immediately scored and participants needed to score ≤80 to be further included in the study. Participants also completed the modified Gait Self Efficacy (mGES) scale (15) and the Fear of Falling Avoidance Behavior Questionnaire (FFABQ) (16). The former asks individuals to rate confidence in their ability to safely complete specific gait tasks (e.g., walk on uneven surfaces or up stairs); the latter addressees the behavioral impact of fear of falling, a correlate of low balance confidence, by evaluating participants' agreement with the statement: “Due to my fear of falling I avoid (activity)…”.

Additional self-reported surveys completed at baseline included the Short-Form Health Survey (SF-36) (17), the Frenchay Activity Index (FAI) (18)*,* and the Well-being scale of the Prosthetic Evaluation Questionnaire (WB-PEQ) (19). We analyzed 3-of-8 dimensions of the SF-36 (role limitation due to physical health problems, social functioning, and role limitation due to emotional problems), which have been recommended for quantifying community participation in LLPUs (20). The FAI measures social participation and daily activities by having participants rate the frequency of participation in activities over the last 3–6 months. For the purposes of this study, we modified the scale to address periods of two months (the intended period between assessments). Finally, the two-item WB-PEQ asks participants to rate their satisfaction and their QoL over the last four weeks using a 10 cm visual analog scale.

After completing surveys, participants performed the Berg Balance Scale (BBS) (21) and the L-test of walking (22) to measure aspects of balance and functional mobility. The BBS requires participants to complete 14 tasks of increasing difficulty beginning with sit-to-stand and progressing to unipedal stance. For the L-test of walking, participants rise from a chair without armrests, walk 3m, turn right, walk 7 m, turn around and trace their path back to the start. The time to complete the task is recorded. Functional tests were assessed by a single, trained investigator blinded to the study purpose or group assignment.

At the conclusion of all baseline assessments, participants were fitted with an activity monitor (Step Watch 4; Modus Health LLC; Seattle WA) that recorded step counts within 10 s epochs using a proprietary algorithm. The monitor was worn on the pylon of the prosthesis (attached via Velcro straps) for one week, during which time it was not necessary to charge the monitor. A minimum of five days with 24 h of recording were necessary to be included in analysis During the week of monitor wear, a researcher called the participant to administer two subscales from the Community Reintegration of Injured Servicemembers (CRIS) (23). We administered the Extent of Participation subscale and the Perceived Limitation subscale, which have been recommended for evaluating community participation in LLPUs (20). The former asks individuals to rate on a 7-point frequency scale how often they engage in certain activities, e.g., “In the past two weeks on average, how often did you engage in recreational activities, not including watching TV?”. The latter uses a 7-point agreement scale to evaluate self-perceived limitations in participation, e.g., “I avoided going to crowded places such as the mall or community gatherings”.

### Randomization

Following baseline screening and assessments, and prior to treatment visit 1, a study team member opened a numbered envelope holding the participants' group assignment, which was stuffed and sealed by a separate investigator uninvolved in recruitment or data collection. Group assignments were determined by a randomization plan created before the start of the study. Block randomization was used with block size of *N* = 4 stratified by cause of amputation (dysvascular vs. all other causes). The logistics of the study necessitated that group assignments be disclosed to study team members prior to treatment visit 1 to coordinate schedules of the participant and the study team members. The physical therapist and CBT counselor were needed for all sessions for participants assigned to the intervention group, whereas only the former was needed for participants assigned to the control group (at-home exercise treatment). The group assignment was not disclosed to the participant until they arrived for treatment visit 1. If participants failed to attend their first session, the randomization plan did not include replacement of the group allocation; the plan continued as if the group assignment was disclosed to the participant to avoid any future bias by study team members during recruitment and screening. The consent documentation did not provide information as to which of the groups was considered the active arm, only that the study was interested in comparing different types of exercises.

### Control group (at-home exercise treatment)

Specific details regarding the control and intervention have been previously reported (12) and a detailed intervention manual is available upon request. Here we briefly review the major components of each. Participants assigned to the control condition attended a single treatment visit where they were provided with a manual that described a series of seated exercises and the schedule for performing the exercises. We intentionally chose seated exercises to minimize any impact on gait and balance, while presenting them as an active treatment that could potentially improve balance confidence. The exercises were adapted from online sources available through the National Institute of Aging and the Center for Disease Control and were to be performed a minimum of 3 times per week for a total of 8 weeks. Different exercises were introduced or dropped throughout the 8 weeks. The physical therapist demonstrated all exercises to the participant and adapted them to the participant as needed. The last page of the manual included a worksheet for participants to mark the days that they performed exercises.

### Intervention group

While the intervention was designed to consist of eight, 90–105 min, weekly sessions that integrated CBT and PT components, some alterations to this scheduling were introduced to minimize participant burden. For example, one participant who had a month-long scheduled vacation received 6 rather than 8 sessions to avoid a one-month delay in study completion. The first session introduced the participant to the treatment rationale, approach, structure and equipment, and ended with a homework assignment connected to the PT and CBT content. Each subsequent session began with a brief initial interaction between the CBT counselor and participant to check in and review weekly homework assignments [e.g., a structured behavioral recording form; see Supplementary information in (12)]. Additional homework components are outlined in Table 1.

**Table 1:** Overview of key activities during the 8-session CBPT intervention.

Session	Key activities	Homework
1	• Introduction to some virtual reality games • Program overview • Introduction to behavioral recording form	• Complete behavioral recording form
2	• Introduction to additional virtual reality games • Review of homework • Develop behavioral goals • Introduction of diaphragmatic breathing	• Complete behavioral recording form • Review diaphragmatic breathing information
3–7	• Virtual reality gaming • Homework review and practice of diaphragmatic breathing • Introduce systematic exposure in the context of reducing fear and changing behavior • Exposure exercise	• Complete behavioral recording form • Practice diaphragmatic breathing • At home exposure exercises
8	• Summarize progression made toward behavioral change goals and in balance and gait • Present strategies for preventing relapse	

Following the check in, participants engaged in a 45–60 min session of virtual reality (VR) gaming using the C-Mill balance suite (Motek Medical, Houten, The Netherlands). Six games targeting the domains of balance (postural responses to self-induced destabilizations or shifts in the center of mass) and gait adaptability (the ability to adjust gait to unpredictable environments) were included. A detailed description of the VR exergames can be found elsewhere (12). Whereas the intervention was designed to include eight, 5-minute blocks of gaming per session, fewer blocks were often included, e.g., due to the need for longer rest periods and/or longer than anticipated check-ins.

During the gaming session the participant wore an overhead harness to prevent a fall to the treadmill surface. The progression of gaming between blocks was guided by a preset progression that was modified based on session-to-session fluctuations in the participants energy, mood and pain. After each block participants reported on their “current level of distress” on a scale of 0 (“no distress”) to 100 (“extreme distress”), as well as their level of stability (assuming the harness was not present) on a scale of 1 (“completely stable as if you were standing or sitting undisturbed on solid ground”) to 10 (“about to fall—extremely challenging, have to stop and/or grab supports to keep balance”) and responses guided the physical therapist's choice to progress gaming; too little distress or instability suggested a need to increase the level of difficulty, and too much indicated a need to reduce the level. The CBT counselor was present throughout the gaming to facilitate integration of PT and CBT techniques. For example, if the CBT counselor identified signs of stress during gaming, they would encourage learned relaxation techniques.

Following the gaming portion of the intervention, the participant completed a short exposure therapy procedure (sessions 2–8 only). Based on review of behavioral data from homework assignments, the physical therapist and CBT counselor structured a low-level exposure exercise to approximate a community-based task that was avoided (or completed, but with significant anxiety) and that was congruent with the participant's functional abilities. For example, a participant who avoided outdoor activities that required walking on uneven surfaces like trails or grass was exposed to walking on the uneven grassy surfaces surrounding the university. Throughout the exposure exercise, the participant reported on distress using the same scale as during gaming. The goal was to observe a reduction in distress with continued exposure, in response to which the degree of exposure would progress. Throughout the exposure exercise, the CBT counselor reinforced the connection between the exercise and the avoided activity. Following the exposure therapy, the CBT counselor further reviewed homework assignments, taught new or reinforced previously learned coping skills (e.g., relaxation strategies) and discussed upcoming assignments. Session activities are summarized in Table 1. While the CBT components were designed to focus on balance-confidence- and/or fear-of-falling-related barriers to community participation, in several cases participants struggled to identify specific barriers in these areas, but did identify other poignant barriers related to their prosthetic device or amputation, e.g., feeling judged for their actions when wearing pants that made their disability “invisible”, or depressive symptoms connected to their amputation. Rather than redirecting conversations to focus exclusively on balance confidence or fear-of-falling, the CBT counselor addressed such issues as they emerged, with the ultimate goal of increasing community participation in mind. This approach reflects how mental health clinicians typically operate in clinical rehabilitation settings.

At the end of each session, after the participant left, the CBT counselor and physical therapist discussed their emerging understanding of the participant's fear hierarchy relating to prosthetic use, and their progress. The goal was to identify the progression of the existing exposure exercise, or to identify a more appropriate exercise for future sessions.

### Follow-up assessments

One week after completion of the treatment (CBPT intervention or at-home exercises), participants returned to the laboratory to repeat all baseline assessments (i.e., post-treatment assessments). Prior to returning, an investigator contacted the participant to conduct the CRIS over the phone. Participants who completed the CBPT intervention also took part in a semi-structured key informant interview with one of the investigators to address their experience with the intervention. Eight- and 16-weeks following the post-treatment assessment, participants were mailed all self-reported outcome measures and were contacted to complete the CRIS. Although the primary interest was on the immediate effects of the intervention (baseline- vs. post-treatment-assessments), the follow-up time points were added to better capture the full extent to which training-induced changes in behavior were incorporated into daily living. Most studies evaluating the effect of multicomponent interventions on balance confidence and related constructs, at least in older adults, report only on the immediate training effects and fail to consider the extent to which benefits remain over time (11).

### Sample size and analysis plan

To test H1–3 we planned to run a mixed effects model with group as the between-subject factor, with time (4 timepoints for H1–H3 and 2 timepoints for H4) as the within-subject factor, and with a random intercept. A significant time × group interaction would demonstrate support of the hypotheses. A priori, we expected to recruit 60 eligible participants (30 intervention and 30 control participants), which, based on a sensitivity analysis, specifying a power of 0.80, an alpha of 0.05, and an average correlation of observations of 0.50, would allow us to detect a small-to-medium effect (f = .152), consistent with the size of effect of multicomponent interventions on balance confidence in older adults (8, 24). However, as a result of significant recruitment challenges, in part due to COVID-19 restrictions, we altered the planned analysis.

Given that detection of a significant interaction requires a considerably larger sample size than is needed to detect a main effect (25), we limited analysis to evaluate main effects that, collectively, we would interpret as evidence supporting an interaction. Specifically, we first considered only baseline and post-treatment data, as this included the largest number of samples, and conducted 2 (group) × 2 (time) repeated measures ANOVAs with self-reported and functional outcomes as the dependent variables. Support of hypotheses would be demonstrated if all of the following criteria were met based on *post-hoc* analyses using LSD corrections: (1) no significant differences between groups at baseline; (2) no significant baseline- vs. post-treatment improvement for the control group; and (3) a significant baseline- vs. post-treatment improvement for the intervention group. If the intervention group scored significantly lower at baseline, then criteria (1) would be altered to “no significant differences between groups post-intervention”; significantly higher scores in the intervention group at baseline would not allow for the type of analysis suggested. Cohen's *d* values are provided for all pairwise comparisons, with the following cutoffs for interpretation: small—0.2 ≤ d < 0.5; medium—0.5 ≤ d < 0.8; large—0.8 ≤ d.

For variables that did not meet criteria 1–3 above (i.e., group-level criteria), we evaluated individual responsiveness by determining whether or not change scores (post-treatment—baseline) exceeded the minimum detectable change (MDC) (see Table A1 in Supplementary Material for justification of MDC). If at least 30% of scores in the CBPT group exceeded MDC (in a positive direction), and this percentage exceeded the similarly calculated percentage for the control-group, then we would also interpret this as initial evidence of an intervention effect (i.e., individual-level criteria).

Finally, for each outcome, paired samples t-tests were used to compare values between 16-week follow-up and post-treatment. The interpretation of these results depended on those from the baseline vs. post-treatment analysis. For outcomes demonstrating an initial effect (baseline vs. post-treatment) of the CBPT intervention, persistence of effects over time would be evidenced by no further change (or continued improvement) in the outcome for the intervention group, and no change in the control group. For outcomes that were not initially affected by the intervention, improvements (16-week follow-up vs. post-treatment) in these outcomes for the intervention group but not the control group would suggest an intervention effect, but only after time; certain behavioral changes may require time to manifest. It should be noted that demonstrating the persistence of effects over time does not imply that the strength of effects was unchanged over time.

Thematic analysis was used to identify themes in key informant interviews. Interviews transcripts were reviewed by a study investigator to develop an initial codebook. Two coders reviewed the codebook with the investigator prior to coding transcripts. One transcript was coded independently by the investigator and coders, who then met to review the transcripts and resolve discrepancies; changes to the codebook were made. This process occurred two more times; the coders then independently coded the remaining transcripts and reviewed their coding with the investigator to resolve discrepancies. From the coding structure, themes were identified.

## Results

We ultimately recruited 22 participants of which 19 were randomized (two failed screening and one was withdrawn for medical reasons between screening and randomization; Figure 1), and of which 10 were assigned to the CBPT intervention group. On average, the 19 randomized participants were older (mean age 62.2 ± 12.9 years), overweight (mean body mass index: 28.7 ± 5.6 kg/m^2^) adults, with comfortable sockets (all but two participants reported socket comfort scores of ≥8-of-10), with most (14-of-19) having traumatic or other non-dysvascular etiology, and with no significant difference in key characteristic between groups (see Table 2 for group-level characteristics and Table 3 for details regarding prosthetic set-ups). Intervention group participants received an average of 7.4 ± 0.9 sessions (3.9 ± 0.5 gaming blocks per session) over an average of 13.4 ± 5.1 weeks. For most outcome measures, data from four control-group participants and nine intervention-group participants were included in analyses evaluating post-treatment effects (i.e., had data at both time points; see Table A2 in Supplementary Material for sample sizes for each analysis and reasons for missing data). When evaluating longer-term effects of the intervention (16-week follow-up), most analyses included data from three control-group participants and six intervention-group participants.

**Table 2:** Characteristics of participants by groups.

	Enrolled			Analyzed		
	CBPT (*n* = 10)	Control (*n* = 9)	*p*-value	CBPT (*n* = 9)	Control (*n* = 4)	*p*-value
Age (yrs)	61.6 ± 15.7	62.9 ± 9.6	0.843	62.4 ± 16.4	60.8 ± 10.3	0.854
BMI (kg/m^2^)	27.0 ± 4.0	30.9 ± 6.7	0.146	27.2 ± 4.2	34.0 ± 8.7	0.076
Time since amputation (yrs)	10.7 ± 11.7	11.9 ± 17.4	0.864	11.4 ± 12.2	22.7 ± 22.2	0.251
Sex						
Male	8	7	0.906	7	2	0.317
Female	2	2		2	2	
Race						
White	9	6	0.466	9	3	0.118
Black	0	1		0	1	
Hispanic	1	1		0	0	
Other	0	1		0	0	
Etiology						
Vascular	3	2	0.701	2	1	0.913
Non-vascular	7	7		7	3	
Assistive device						
Yes	5	2	0.160	5	0	0.057
No	5	8		4	4	
Falls in prior year						
0	5	4	**0**.**006**	5	3	0.197
1	5	0		4	0	
2+	0	5		0	1	

Bold values indicate *p* < 0.05.

**Table 3:** Prosthetic set-up of randomized participants.

Group	ID	Prosthetic Set-up
C	1	Bionx, empower foot; total contact socket; vacuum suspension with harmony
I	2	Flexfoot; total contact w/polyethylene inner socket; mechanical pump (vacuum) with gel suspension sleeve
C	3	Alpha silicone liner; suspension sleeve
I	4	RUSH foot; TSB socket with suction vacuum
C	5	Dynamic Response foot, TSB socket with pin lock suspension; 1 ply sock with silver sheath inner against skin
I	6	College Park foot multiaxial; total contact socket; flexible plastic inner medium plus gel liner; sleeve suspension; 6 mm sock
I	7	dynamic response foot; currently in temporary socket; suction seal in suspension (iceross seal-in); light weight soft sock
I	8	Elation foot; Ossur gel liner; pin suspension
C	10	Ossur running leg for daily use; TSB socket; sleeve suspension with expulsion valve on socket
C	11	total contact socket with gel liner; pin lock suspension; 5 ply socks with and distal cup
I	12	Dynamic Response Low Profile foot; TSB socket; vacuum suspension with sleeve (Harmony P4); 3mm gel liner
I	13	Ossur low profile Dynamic Response Split Toe foot; TSB socket; vacuum heel pump system with silicone gel liner silicone
C	15	Ossur Dynamic Response foot; suction with sleeve suspension; Silver 1ply sock against skin (gel liner)
C	16	Ossur Dynamic Response/UNITY foot; total contact socket with gel liner system; suction with suspension sleeve
C	17	Not collected
I	18	Ossur Pro-flex XC, category 7 foot (suspension/liner not noted)
I	20	non articulating, carbon graphite foot; plastic inner socket; sleeve suspension
I	21	Kinterra DF/PF motion carbon fiber foot; carbon fiber socket with soft silicone plastic inner liner and alpha silicone liner; sleeve suspension with supra condular clip
C	22	carbon graphite foot; silicone liner with pin suspension

Group: C- Control, I—Intervention.

### Balance confidence and related constructs

**Figure 2:**
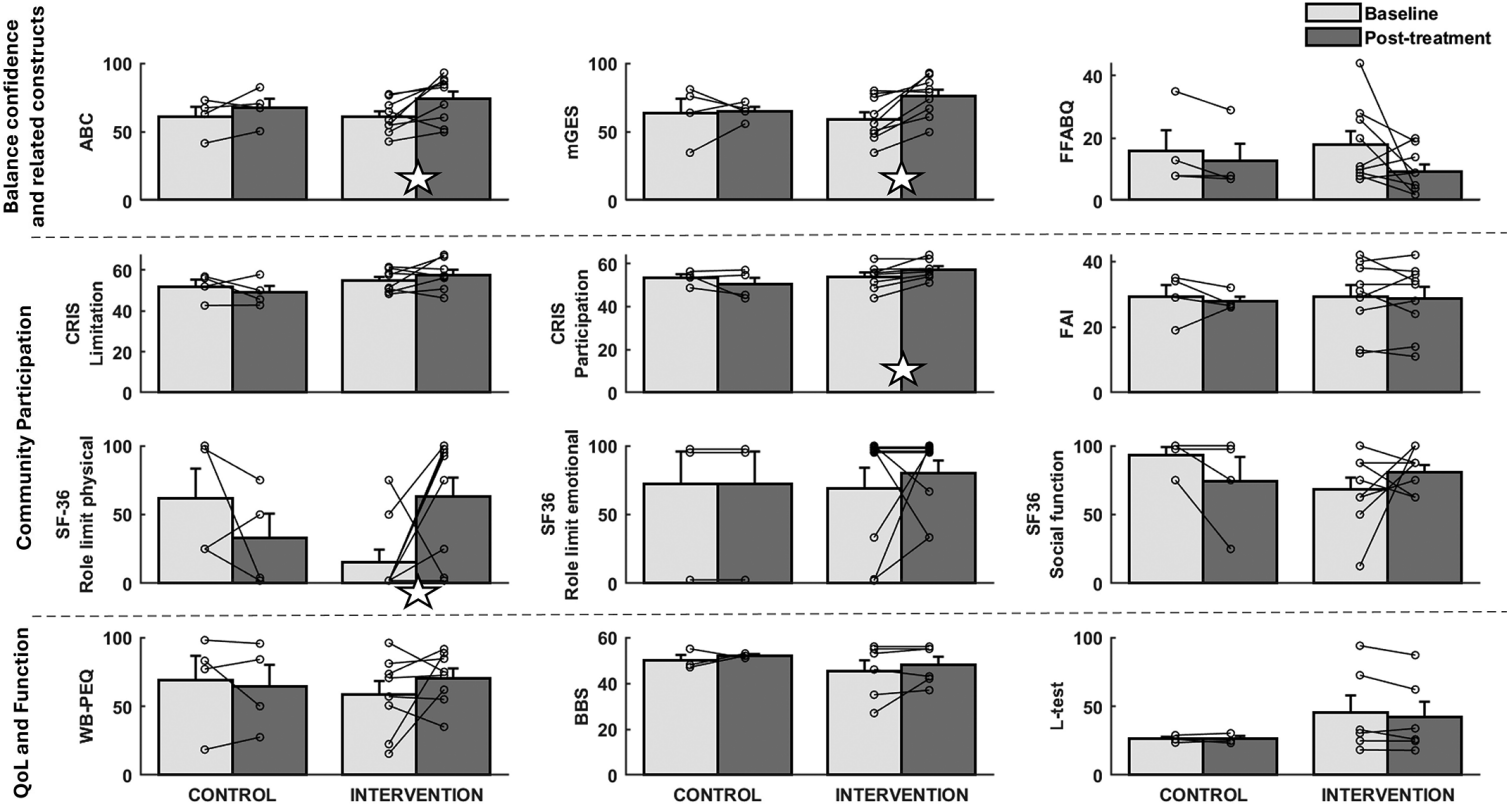
Visual representation of individual and group data for all outcomes grouped by domain. Bar plots show group-level means and standard errors for each outcome with light grey bars for the baseline values and dark grey for post-treatment. The individual data points that contribute to the group data are displayed. Note that steps/day is not included under community participation and that quality of life and function are grouped together for ease of visualization. ABC, activity-specific balance confidence; FFABQ, fear of falling avoidance behavior questionnaire; mGES, modified gait efficacy scale; CRIS, community reintegration of injured servicemembers scale; SF-36, 36 question short form; FAI, frenchay activity index; PEQ-WB, well-being scale of the prosthetic evaluation questionnaire; BBS, berg balance scale; open white star indicates *p* ≤ 0.05.

Whereas there was no difference in balance confidence (ABC scale score) between groups at baseline (*p* = 0.974), there was a significant increase from baseline to post-treatment for the intervention group (*p* = 0.033) but not for the control group (*p* = 0.308), with changes in the CBPT group indicative of a strong intervention effect (Cohen's d = 0.86; Tables 4, 5; Figure 2). There was no significant change in ABC scores for participants in either group between post-treatment and 16-week follow-up (all *p* > 0.17).

**Table 4:** Group averages (mean ± SD) and *p*-values for outcome measures by timepoint for those with repeated data.

Scale	Time point	Control	CBPT	*p*-values
Balance confidence and related constructs				
ABC scale	baseline	61.4 ± 13.7	61.2 ± 11.8	*p* = 0.974
	post-treatment	67.9 ± 13.2	74.2 ± 16.5	*p* = 0.380—control: post vs. base ***p* = 0.018—CBPT: post vs. base**
	16-week follow-up	65.6 ± 15.2	72.0 ± 19.9	*p* = 0.480—control: 16-week vs. post *p* = 0.175—CBPT: 16-week vs. post
mGES	baseline	64.0 ± 20.6	59.2 ± 15.8	*p* = 0.653
	post-treatment	65.0 ± 6.7	75.9 ± 14.4	*p* = 0.887—control: base vs. post ***p* = 0.004—CBPT: base vs. post**
	16-week follow-up	65.7 ± 14.6	73.5 ± 23.1	*p* = 0.899—control: 16-week vs. post *p* = 0.212—CBPT: 16-week vs. post
FFABQ	baseline	16.0 ± 12.8	18.1 ± 12.5	*p* = 0.786
	post-treatment	12.8 ± 10.8	9.3 ± 6.9	*p* = 0.135—control: post vs. base *p* = 0.063—CBPT: post vs. base
	16-week follow-up	15.3 ± 16.7	10.8 ± 10.4	*p* = 0.843—control: 16-week vs. post *p* = 0.595—CBPT: 16-week vs. post
Community participation				
CRIS Limitation	baseline	51.9 ± 6.5	54.7 ± 5.4	*p* = 0.433
	post-treatment	49.1 ± 6.5	57.7 ± 7.2	*p* = 0.455—control: post vs. base *p* = 0.278—CBPT: post vs. base
	16-week follow-up	47.4 ± 11.5	58.4 ± 4.7	*p* = 0.770—control: 16-week vs. post *p* = 0.393—CBPT: 16-week vs. post
CRIS Participation	baseline	53.2 ± 3.2	53.7 ± 5.6	*p* = 0.874
	post-treatment	50.2 ± 6.6	57.2 ± 4.2	*p* = 0.161—control: post vs. base ***p* = 0.034—CBPT: post vs. base**
	16-week follow-up	48.4 ± 9.5	56.9 ± 3.2	*p* = 0.921—control: 16-week vs. post *p* = 0.969—CBPT: 16-week vs. post
FAI	baseline	29.3 ± 7.3	29.2 ± 10.9	*p* = 0.996
	post-treatment	27.8 ± 2.9	28.8 ± 10.6	*p* = 0.570—control: post vs. base *p* = 0.800—CBPT: post vs. base
	16-week follow-up	28.7 ± 3.2	29.5 ± 10.7	*p* = 0.728—control: 16-week vs. post *p* = 0.249—CBPT: 16-week vs. post
SF-36—Role Limit Physical	baseline	62.5 ± 43.3	13.9 ± 28.3	***p* = 0.032**
	post-treatment	31.3 ± 37.5	63.9 ± 43.5	*p* = 0.295—control: post vs. base ***p* = 0.023—CBPT: post vs. base**
	16-week follow-up	50.0 ± 50.0	40.0 ± 45.4	*p* = 0.423—control: 16-week vs. post *p* = 1.000—CBPT: 16-week vs. post
SF-36—Role Limit Emotion	baseline	75.0 ± 50.0	70.4 ± 45.5	*p* = 0.872
	post-treatment	75.0 ± 50.0	81.5 ± 29.4	*p* = 1.000—control: post vs. base *p* = 0.451—CBPT: post vs. base
	16-week follow-up	66.7 ± 57.7	86.7 ± 29.8	*p* = 1.000—control: 16-week vs. post *p* = 1.000—CBPT: 16-week vs. post
SF-36—Social Function	baseline	93.8 ± 12.5	68.0 ± 25.9	*p* = 0.090
	post-treatment	75.0 ± 35.4	80.6 ± 15.5	*p* = 0.281—control: post vs. base *p* = 0.281- CBPT: post vs. base
	16-week follow-up	75.0 ± 33.1	72.5 ± 22.4	*p* = 0.184—control: 16-week vs. post *p* = 1.000—CBPT: 16-week vs. post
Steps/day	baseline	3,802.1 ± 2,208.2	5,475.1 ± 2,997.1	*p* = 0.415
	post-treatment	3,488.4 ± 2,263.4	4,479.2 ± 2,830.2	*p* = 0.625—control: post vs. base ***p* = 0.039—CBPT: post vs. base**
	16-week follow-up	3,242.2 ± 1,766.2	5,536.3 ± 2,439.3	*p* = 0.642—control: 16-week vs. post *p* = 0.671—CBPT: 16-week vs. post
Quality of life				
WB-PEQ	baseline	69.1 ± 34.9	58.3 ± 28.0	*p* = 0.572
	post-treatment	64.3 ± 31.2	70.5 ± 19.1	*p* = 0.729—control: post vs. base *p* = 0.236—CBPT: post vs. base
	16-week follow-up	61.2 ± 46.2	71.1 ± 24.4	*p* = 0.658—control: 16-week vs. post *p* = 0.167—CBPT: 16-week vs. post
Function				
BBS	baseline	50.0 ± 4.4	45.3 ± 11.9	*p* = 0.543
	post-treatment	52.0 ± 1.0	48.0 ± 8.3	*p* = 0.583—control: post vs. base *p* = 0.314—CBPT: post vs. base
L-Test	baseline	26.2 ± 2.7	45.5 ± 30.4	*p* = 0.323
	post-treatment	26.2 ± 3.7	41.9 ± 27.0	*p* = 0.991—control: post vs. base *p* = 0.101—CBPT: post vs. base

Bold values indicate *p* < 0.05.

ABC, activity-specific balance confidence; FFABQ, fear of falling avoidance behavior questionnaire; mGES, modified gait efficacy scale; CRIS, community reintegration of injured servicemembers scale; SF-36, 36 question short form; FAI, frenchay activity index; PEQ-WB, well-being scale of the prosthetic evaluation questionnaire; BBS, berg balance scale; see Tables A1 and A2 in Supplementary Material for sample sizes for each comparison and for interpretation of values– i.e., whether higher or lower values indicate improvements. Post-treatment data was separately compared with both baseline and 16-week follow-up data, with different sample sizes in each comparison. 16-week follow-up includes only a subgroup of participants from baseline such that direct comparison between the two should be cautiously interpreted. Within the table, descriptive data for post-treatment reflects the data that was directly compared with baseline.

**Table 5:** Mean within subject changes in outcome measures by group and time with effect sizes.

	Control		CBT	
	Post-treatment vs. baseline	16-week vs. post-treatment	post-treatment vs. baseline	16-week vs. post-treatment
Balance confidence and related constructs				
ABC scale				
Change score	6.4 ± 10.5	−2.3 ± 4.6	13.0 ± 15.2	−3.7 ± 5.8
Cohen's d (95% CI)	0.61 (−0.51–1.66)	−0.50 (−1.67–0.77)	**0.86 (0.07–1.61)**	−0.64 (−1.51–0.27)
mGES				
Change score	1.0 ± 16.7	0.7 ± 8.0	16.7 ± 12.4	−5.5 ± 9.4
Cohen's d (95% CI)	0.06 (−0.93–1.04)	0.08 (−1.06–1.21)	**1.34 (0.41–2.24)**	−0.58 (−1.44–0.31)
FFABQ				
Change score	−3.3 ± 3.2	0.7 ± 5.1	−8.8 ± 14.8	2.5 ± 10.8
Cohen's d (95% CI)	−1.02 (−2.22–0.28)	0.13 (−1.02–1.25)	−0.59 (−1.29–0.14)	0.23 (−0.59–1.03)
Community participation				
CRIS limitation				
Change score	−2.8 ± 7.2	−1.3 ± 6.9	2.9 ± 7.2	−1.1 ± 2.6
Cohen's d (95% CI)	−0.39 (−1.38–0.66)	−0.19 (−1.31–0.97)	0.41 (−0.33–1.12)	−0.42 (−1.32–0.52)
CRIS participation				
Change score	−3.0 ± 5.4	−0.4 ± 5.6	3.5 ± 3.2	0.02 ± 1.1
Cohen's d (95% CI)	−0.56 (−1.59–0.55)	−0.07 (−1.19–1.08)	**1.09 (0.18–1.95)**	0.02 (−0.86–0.89)
FAI				
Change score	−1.5 ± 6.0	0.7 ± 2.9	−0.4 ± 4.8	1.3 ± 2.5
Cohen's d (95% CI)	−0.25 (−1.23–0.77)	0.23 (−0.95–1.36)	−0.09 (−0.75–0.57)	0.53 (−0.35–1.37)
SF-36 physical				
Change score	−31.3 ± 51.5	33.3 ± 57.7	50.0 ± 58.6	0.0 ± 58.6
Cohen's d (95% CI)	−0.61 (−1.65–0.52)	0.58 (−0.72–1.77)	**0.85 (0.06–1.61)**	0.00 (−0.88–0.8)
SF-36 emotional				
Change score	0.0 ± 0.0	0.0 ± 0.0	11.1 ± 50.0	0.0 ± 0.0
Cohen's d (95% CI)	--	--	0.22 (−0.45–0.89)	--
SF-36 Social				
Change score	−18.8 ± 23.9	8.3 ± 7.2	12.5 ± 35.9	0.0 ± 12.5
Cohen's d (95% CI)	−0.78 (−1,88–0.41)	1.16 (−0.44–2.64)	0.35 (−0.34–1.01)	0.00 (−0.88–0.88)
Steps per day				
Change score	−313.7 ± 1,330.6	−588.7 ± 1,322.8	−995.9 ± 964.5	358.9 ± 1,946.9
Cohen's d (95% CI)	−0.24 (−1.36–0.94)	−0.45 (−1.85–1.11)	**−1.03 (−1.94–−0.07)**	0.18 (−0.63–0.98)
Quality of life				
WB-PEQ				
Change score	−4.9 ± 19.4	7.3 ± 24.7	12.2 ± 30.1	7.4 ± 11.3
Cohen's d (95% CI)	−0.25 (−1.23–0.77)	0.30 (−0.90–1.43)	0.40 (−0.33–1.11)	0.66 (−0.26–1.53)
Function				
BBS				
Change score	2.0 ± 5.2	--	2.7 ± 6.3	--
Cohen's d (95% CI)	0.39 (−0.84–1.53)		0.42 (−0.44–1.24)	
L-test (s)				
Change score	0.0 ± 2.4	--	−3.6 ± 6.3	--
Cohen's d (95% CI)	0.01 (−1.12–1.14)		−0.68 (−1.55–0.25)	

Bold values indicate *p* < 0.05.

ABC, activity-specific balance confidence; FFABQ, fear of falling avoidance behavior questionnaire; mGES, modified gait efficacy scale; CRIS, community reintegration of injured servicemembers scale; SF-36, 36 question Short form; FAI, frenchay activity index; WB-PEQ, well-being scale of the prosthetic evaluation questionnaire; BBS, berg balance scale.

The effects of the intervention on gait self-efficacy mirrored those for balance confidence. Whereas scores on the modified Gait Efficacy Scale (mGES) were not significantly different between groups at baseline (*p* = 0.653), mGES scores significantly increased for those who received the CBPT intervention (*p* = 0.004 baseline vs. post-treatment) but did not significantly change for those in the control group (*p* = 0.912). There was no significant change in mGES scores for participants in either group between post-treatment and 16-week follow-up (all *p* > 0.21).

**Figure 3:**
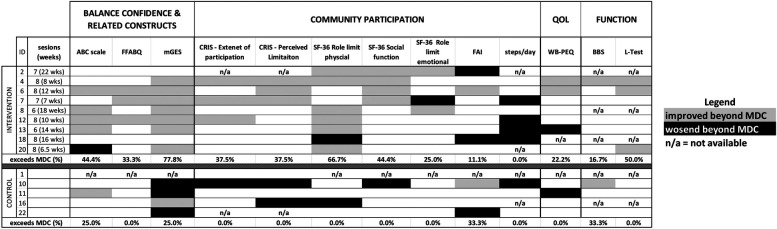
Individual responsiveness (baseline vs. post-treatment) for all outcomes. For each outcome participant change scores were color coded to express the change relative to the detectable change (MDC), or minimum clinically important difference (MCID) for L-test only, Grey boxes indicate that changes were in “positive” direction, i.e., the outcome improved and the change was greater than MDC (or MCID); black boxes indicate meaningful changes in the opposite direction, i.e., the metric worsened; n/a indicates that data was not available. The final row of data for each group summarizes the percentage of participants with improvements above MDC/MCID. Individual-level responsiveness is evidenced by a value of 30% or higher in the CBPT group that is also higher than the value for the control group. Eight of the outcome measures demonstrated individual-level responsiveness, four of which did not show group-level effects. ABC, activity-specific balance confidence; FFABQ, fear of falling avoidance behavior questionnaire; mGES, modified gait efficacy scale; CRIS, community reintegration of injured servicemembers scale; SF-36, 36 question short form; FAI, frenchay activity index; PEQ-WB, well-being scale of the prosthetic evaluation questionnaire; BBS, berg balance scale.

Activity avoidance due to fear of falling, as measured by the Fear of Falling Avoidance Behavior Questionnaire (FFABQ), was not significantly different between groups at baseline (*p* = 0.786), and did not significantly improve post-treatment for those who received the CBPT intervention (*p* = 0.113). However, 33% of participants in the intervention group (3-of-9) reported changes in FFABQ above the MDC (Figure 3).

### Community participation

Among the seven measures used to assess community participation, four met either the group- or individual-level criteria for an intervention effect. Two variables—the CRIS Extent of participation scale and the SF-36 role limitations due to physical health scale—met the group-level criteria to suggest an intervention effect. CRIS—Extent of Participation was not different between groups at baseline (*p* = 0.874), and there was a significant baseline vs. post-treatment increase in the scale for the CBPT group (*p* = 0.018) but not for the control group (*p* = 0.346). With regard to the SF-36 role limitation due to physical health, participants in the CBPT group had significantly lower scores than those in the control group at baseline (62.5 ± 43.3 for control group at baseline vs. 13.9 ± 28.3 for intervention group, *p* = 0.032). However, between-group differences were no longer significant at post-treatment (*p* = 0.222), with scores significantly improving from baseline for the CBPT group (*p* = 0.023). The other five community participation outcomes did not significantly increase from baseline to post-treatment for the CBPT group (all *p*-values > 0.28). However, two of these measures—the CRIS Perceived Limitation subscale and the SF-36 social function subscale—met the individual-level criteria for an intervention effect with 38% and 44% of CBPT participants, respectively, showing improvements beyond MDCs.

Within the domain of community participation, physical activity (measured as steps per day) showed a group-level effect in the opposite direction to that expected. Whereas there was no significant difference in activity between groups at baseline (*p* = 0.415) there was a significant post-treatment reduction in activity for the CBPT group (*p* = 0.039) that was absent from the control group (*p* = 0.625).

### Functional mobility and quality of life

There were no significant group-level effects of the CBPT intervention from baseline to post-treatment on any of the functional mobility outcomes or QoL (all *p*-values > 0.10). Evidence of an individual-level effect on walking ability (L-test) was observed, with 50% of CBPT participants (3-of-6) demonstrating changes that exceeded the MDC (Table 4).

### Longer term effects of intervention

For the four variables demonstrating a significant group-level effect of the intervention, there were no significant changes over the 16-week follow-up for participants in either group (all *p*-values > 0.17; Table 4). There were no cases in which variables that were not initially impacted by the intervention showed an effect at 16 weeks.

### Key informant interviews

Of the 10 CBPT participants, 7 (70%) completed the key informant interview. Three main themes were identified: (1) Participants benefited from the CBPT; (2) Participants recommended improvements to the CBPT intervention; and (3) Participants were unique. Table 6 includes the themes, subthemes, codes that connected to the themes, and sample quotes that illustrate the themes. All 7 participants had content coded for each theme.

**Table 6:** Themes, codes and sample quotes from key informant interviews (*n* = 7).

Themes, subthemes	Codes	Sample quotes (Participant ID #)
Intervention benefits *Enjoyment*	Physical improvements Psychological improvements Interventionists cared Enjoyed treadmill game Enjoyed physical therapy Enjoyed integration of physical therapy and cognitive behavioral therapy	I was sitting around more. You know, and I feel like I get out, and I can do more, you know, even walking to the garden now without a cane, you know, and stuff like that. So, yeah, that's, that was the main thing (DOD012). I really, I wish you could, you guys could just know how I felt and what I couldn't do and what I could do. It's like, it's, it's like a rush. I just feel more positive, you know, my life's better. If anything, this has made my life better mentally and physically. That's all I can say. Yeah, it truly has, you know, you guys might think, yeah, okay, we help the guy out. But no, you did more than that. And that's why like, [redacted], and my friends and family just won't believe that, you know, they're also happy for me. They're doing that for you. And I go, Yeah. Yeah, that's a good thing (DOD006). You know, we're talking about the confidence of like, not losing your balance or, you know, I think, I think having those two components, the games, and then you know, the kind of like the psychologist, I think it that meets the goal really well. So, I think if we were to just do the games Yeah, they'd be fun. I don't know if I would have gotten as much out of this personally because, you know, you guys did help talk me through a few things (DOD013).
Recommended improvements *More guidance, feedback*	Study concerns Program challenges Treadmill game malfunctioned More information provided Pain is a barrier to activity Homework problems More sessions needed	I know it puts a burden on everybody to drive here and get here on time and so forth (DOD004). I think that we talked [the homework] through and I understood it, and if you were to read this to me it doesn't make sense to someone who would pick it up and read it, it makes sense to me because we went through and explained it, you know what I'm saying? (DOD007) but really it was just the pain and the risk of causing skin damage that was the only thing holding me back because it just happened so often. But otherwise, everything else was positive about that. (DOD008)
Unique participants	Motivated participants	Well, when I got the notice, I was excited about it, because I wanted to get better. (DOD020)

## Discussion

The purpose of this study was to conduct a randomized controlled trial to evaluate the initial effects of a novel CBPT intervention on four domains: (i) balance confidence, (ii) community participation, (iii) functional mobility and (iv) QoL**.** Overall, results demonstrate the initial efficacy of the CBPT intervention, with at least one outcome in 3-of-4 domains (balance confidence, community participation and functional mobility) showing significant group- or individual-level effects, and participants reporting improvements as highlighted in the qualitative data analysis. For the final domain, QoL, only 22% of respondents showed individual responsiveness (changes > MDC) with an overall weak intervention effect (Cohen's *d* = 0.40; Table 5). Collectively, the findings support the promise of multidisciplinary interventions for inducing lasting improvements in balance confidence and related domains that can impact mental and physical health in LLPUs. To the best of our knowledge the current study is the first to evaluate an intervention to specifically target balance confidence in LLPUs.

Integrating CBT approaches with exercise-based interventions could be an ideal approach to target balance confidence and related constructs in LLPUs. Multidisciplinary rehabilitation teams with varying skillsets may be better equipped to identify true physical limitations vs. self-imposed ones due to low balance confidence. These teams are well-suited to tailor the intervention to focus on activities connected to both low balance confidence and to the patient's goals. Moreover, use of a multidisciplinary team to address the physical and psychological contributors to behavior has a strong theoretical basis. Indeed, Bandura's theory suggests that one's perception of their ability to perform a task (i.e., self-efficacy, assessed here as balance confidence) is at least as important to predicting behavior as is having the necessary skill to perform the tasks (26, 27). As an example of this concept, one can imagine a case where, despite having the skill to ambulate safely, an LLPU may limit their walking if they lack balance confidence. The need for a multidisciplinary approach is further highlighted by a 2022 systematic review that reported no significant effect of interventions targeting only physical function (i.e., balance and gait exercises) on balance confidence in LLPUs (28). Relatedly, while componentry (29, 30) or suspension (31) has the potential to improve confidence, changing prosthetic factors alone seemingly addresses only the impact of physical ability on balance confidence, which may limit long-term impact. Moreover, technologies may be intolerable or cost-prohibitive.

Interventions that simultaneously increase both balance confidence and community participation may collectively contribute to higher QoL. Although greater social engagement, reduced isolation, depression and anxiety may collectively contribute to higher QoL (32), there was no significant effect of the CBPT intervention on QoL. The absence of an intervention effect on QoL is surprising given meaningful, individual-level improvements in functional mobility (L-test); higher mobility (L-test scores) has been shown to associate with QoL (WB-PEQ scores) in prosthesis users (33). The absence of meaningful changes in QoL may result from the small sample, which does not allow for consideration of a number of amputation-specific characteristics, such as residual limb health, time since amputation, or prosthetic fit that can all influence QoL (5).

It is also surprising that physical activity decreased, rather than increased, following the intervention. Intervention participants may have changed their activity patterns based on a better understanding of their actual abilities following the intervention. Regardless, the absence of an intervention-induced increase in steps/day may be consistent with the extant literature. A systematic review on the effect of behavioral interventions on physical activity in LLPUs reported mixed effects of these interventions (across relatively few studies) (34). The authors suggested that simplistic assessments of activity, as summarized by steps/day, may not be sensitive to changes induced in LLPUs following behavioral interventions. Still, the observed effect size for the intervention on physical activity (Cohen's d = −1.03; Table 5) is particularly noteworthy. The observed reductions of >1,400 step/day in 4-of-7 participants in the CBPT group (Figure 3) exceed the minimum clinically important differences (MCIDs) reported for other populations undergoing exercise interventions (e.g., 427/day in patients with chronic obstructive pulmonary disease or 1,211 steps/day in patients with peripheral artery disease (35, 36)). Alternative analyses of activity, e.g., ones that account for day-to-day variations in intensity, frequency and possibly location of activity, and which may also account for seasonal fluctuations (37), may provide more accurate and detailed information for future studies attempting to make long-lasting changes in physical activity.

A final surprising observation was the stronger effect of the intervention on gait self-efficacy than on balance confidence (Cohen's d = 0.086 vs. 1.34; Table 5), despite the former not being targeted by the intervention (although intervention sessions included a two-minute warm-up walk and, possibly, one game that targeted gait adaptability). Whereas balance confidence was assessed using a scale with somewhat broad items, the gait-efficacy scale (mGES) is narrower in scope and focuses only on evaluating confidence in one's ability to walk under varied environmental conditions, which may be more relevant to understanding mobility in terms of activities of daily living (15). Given the broad intervention goal of positively affecting daily behaviors, this surprising observation is encouraging. In addition, improved gait self-efficacy may have positive effects on health and function for the LLPUs, potentially independent from those observed by improving balance confidence. For example, a study on older adults by Rosengren et al. reported that gait efficacy had a significant effect on gait speed (38), an important indicator of morbidity and mortality in older adults (39). Moreover, higher gait self-efficacy has been associated with fewer falls (40) and with higher daily step counts (41) in various patient groups. While, to the best of our knowledge, gait self-efficacy has received little-to-no consideration in the prosthetics literature, it may represent an important construct to consider in behavioral interventions for LLPUs, and one that warrants further evaluation in this cohort.

There are several limitations in the current study that should be considered. First, no attentional control was included, such that effects of the intervention could reflect the fact that the CBPT groups received up to eight face-to-face sessions compared to a single session for the control group. Limited attention in the control group could also contribute to the apparently higher loss to follow-up. Second, the observed efficacy of the intervention may have been impacted (positively or negatively) by protocol deviations described (e.g., not all participants received 8 sessions, and the duration of the intervention varied considerably). However, 8 sessions may not be needed for individuals to show meaningful improvements. For example, two participants who completed only 6 sessions show meaningful improvement in a similar, if not greater, number of outcomes compared to those who completed 8 sessions (Figure 3). Future work may consider introducing a stepped care approach where intervention continues/increases for those who do not experience gains within a specified time frame. A third limitation relates to generalizability. The findings are currently applicable only to patients with transtibial amputations, although balance confidence levels may be similar in those with transfemoral amputations (2). While findings also only apply to chronic prosthesis users, one might expect even stronger effects in acute care (42). Participants were also highly motivated to improve (Table 6); patients with lower motivation may be less likely to engage in the intervention. A final limitation relates to the small sample size, which did not allow for conducting the proposed statistical analyses. However, individual responsiveness helps to provide additional support for the initial efficacy of the intervention. For example, 8-of-9 participants in the CBPT group showed changes that were above MDC in at least 3 outcome measures considered. Only one participant (subject 18) failed to show any positive changes beyond MDC magnitude, which could reflect yet-to-be-determined, unique attributes of the individual.

In conclusion, our novel intervention that integrates physical therapy exercises with CBT strategies to address physical underpinnings and maladaptive cognitions around the construct of balance confidence has the potential to meaningfully improve balance and walking confidence, as well as community participation. Future efforts should work to implement a similar study design on a larger cohort, focusing on those outcomes showing the largest effects herein, and to understand and address barriers related to translation into clinical settings.

## Data Availability

The raw data supporting the conclusions of this article will be made available by the authors, without undue reservation.

## Author's note

The content does not reflect positions of the U.S. Department of Veterans Affairs or the U.S. Government.
