# Supplementary material for: Mixed methods analysis of an interdisciplinary intervention to promote balance confidence in lower limb prosthesis users
Source: Front Rehabil Sci. 2025 Sep 1;6:1626051. doi: 10.3389/fresc.2025.1626051 (PMC12434030; doi:10.3389/fresc.2025.1626051)
Supplement: Supplementary file 1 [file Table1.docx]

**APPENDIX**

**Table A1. Description and properties of assessment tools**

| **Domain** | **Scale** | **Survey/Tool Information** | **Psychometrics** | **MCD/MCID** |
| --- | --- | --- | --- | --- |
| Balance Confidence and related constructs | ABC scale | Individuals rate their level of confidence in completing 16 complex functional tasks without losing balance [14]. Items scored -0-100% and then averaged; higher scores indicate greater confidence | strong reliability (ICC=0.91), internal consistency (Cronbach α = 0.95), and good construct validity in LLPUs (1) | In LLPUS - MDC with a 50% confidence - of 0.58 on a 5-point scale; this equates to MDC of 11.6 on 100 point scale (95%) (2) |
|  | mGES | A measure of self-efficacy specific to gait; ten items scored 0-10 with sum reported; higher scores indicate greater gait self-efficacy | good psychometric properties (3-5) | In older adults - MDC with a 95% confidence calculated from reported SEM - equates to 7.2 (3) |
|  | FFABQ | 14 items, each worded to be consistent with the International Classification of Functioning and Health (ICF) model of activity limitation and participation restriction (6). Sample items include: walking on uneven surface; walking in dimly lit unfamiliar places; items scored from 0-4 with higher total scores indicating greater avoidance | Psychometric properties in older adults with a variety of balance impairment, which included some LLPUs, are good-to-excellent (7); unfamiliar with any prior studies utilizing FFABQ exclusively on LLPUs. | In older adults with balance impairments - MDC with a 95% confidence - 14.7 (7) |
| Community participation | SF-36 | consists of 36 questions that measure eight dimensions of health-related quality of life; higher scores indicate greater QoL | Role limited physical subscale in a veteran with LLPUs specific version of eth SF-36 has good internal validity (ICC=0.81) (8), | For SF-36 Role Limit Physical - In Veterans who are LLPUs - MDC with a 90% confidence interval - 26.3 (8); For SF-36 Social Functioning – In patients with Parkinson’s Disease - MDC with a 95% confidence – 29 (9); For SF-36 Role Limit Emotional – In patients with Parkinson’s disease - MDC with a 95% confidence - 45 (9) |
|  | FAI | 15 items spanning three dimensions - domestic/chores, work/leisure and outdoor activities; items rated on 4-point scale and total score is taken; higher scores indicate greater social participation | a valid and reliable measure among LLPU (ICC=0.79) (10). | In patients with chronic strike - MDC with 95% confidence - 6.7 (11) |
|  | CRIS | Addresses the nine chapters of the International Classification of Functioning and Disability Health that deal with activity and participation (12); only two-of-three subscales were provided; Initially designed for interviewing with each CRIS subscale requiring about 15 minutes to administer (12) (although a validated computer adaptive version has been developed (13, 14)); higher scores indicate greater participation/less limitation | CRIS has strong reliability, conceptual integrity, and construct validity (12) | For Extent of participation Scale – In patients with a wide range of polytraumatic injuries including LLPUs - MDC with a 90% confidence - 4.74 (15); For Perceived Limitation Scale – In patients with a wide range of polytraumatic injuries including LLPUs - MDC with a 90% confidence - 5.79 (15). |
|  | Steps/day via Step Watch | Monitor worn on the distal end of the pylon that tracks step count in bins of set duration; no well-established protocol for accepting or rejecting data; newer models with proprietary algorithms require 5 days of valid data (REF), which was also the threshold used here | Monitor validated to reliability and accurately detect step counts in LLPUs (16, 17) | In patients with peripheral arterial diseases undergoing exercises interventions - moderately strong effect of intervention on steps/day – changes of 1392 steps (18) |
| QoL | WB-PEQ | Part of the larger Prosthetic Evaluation Questionnaire, the WB-PEQ includes 2 scored items; each item is scored from 0-100 (based on millimeters along the scale) and the average is taken, with higher numbers indicating greater quality of life; considered a simple-to-use assessment of QoL as participants must only consider the construct as unidimensional. | good consistency (Cronbach α = 0.83) and reliability (ICC=0.89) (19). | In LLPUs - MDC with a 90% confidence - 1.4 points on 7-point scale equates to 20 points on 100 point scale (8) |
| Functional mobility | BBS | 14 items performed by an individual and scored by a PT on a scale of 0-4 with higher scores indicating better balance. | validated for persons with lower limb amputation (20) | In adults with balance deficits - MDC with a 95% confidence - 6.2 (21) |
|  | L-Test | Developed to avoid ceiling effects of timed-up-and-go (TUG) in high functioning LLPUs; time to complete the walk is recorded; | strong reliability (ICC=0.98) and validity (r=0.93 with TUG) (22). | In LLPUs - MCID of 4.5 s (23) |

**Table A2. Sample sizes for each outcome by group and time, corresponding only to participants entered into repeated measures analysis, i.e. with data at both time points**

| **Outcome** | **Group** | **Sample Size Baseline & Post-Treatment** | **Notes** | **Sample Size Post-Treatment & 16-Weeks** | **Notes** |
| --- | --- | --- | --- | --- | --- |
| ABC scale | Intervention | 9 | 1a,2 | 6 | 1c,2,11 |
|  | Control | 4 | 6,7,8a | 3 | 6,7,8a,9b |
| mGES | Intervention | 9 | 1a,2 | 6 | 1c,2,11 |
|  | Control | 4 | 6,7,8a | 3 | 6,7,8a,9b |
| FFABQ | Intervention | 9 | 1a,2 | 6 | 1c,2,11 |
|  | Control | 4 | 6,7,8a | 3 | 6,7,8a,9b |
| CRIS  (both subscales) | Intervention | 8 | 1b,2 | 5 | 1c,2,11,12 |
|  | Control | 4 | 6,7,8b,9a | 3 | 6,7,8b,9b,10b |
| FAI | Intervention | 9 | 1a,2 | 6 | 1c,2,11 |
|  | Control | 4 | 6,7,8a | 3 | 6,7,8a,9b |
| SF36  (all subscales) | Intervention | 9 | 1a,2 | 5 | 1c,2,11,12 |
|  | Control | 4 | 6,7,8a | 3 | 6,7,8a,9b |
| Steps/day | Intervention | 7 | 1b,2,5 | 6 | 1c,2,5,11 |
|  | Control | 3 | 6,7,8a,10a | 2 | 6,7,8a,9b,10b |
| WB-PEQ | Intervention | 8 | 1a,2,3 | 6 | 1c,2,11 |
|  | Control | 4 | 6,7,8a | 3 | 6,7,8a,9b |
| BBS | Intervention | 6 | 1b,2,3b,4 | -- | -- |
|  | Control | 3 | 6,7,8a,10a | -- | -- |
| L-test | Intervention | 6 | 1b,2,3b,4 | -- | -- |
|  | Control | 3 | 6,7,8a,10a | -- | -- |

Notes:

1a - Includes data carried over from session 4 for one participant (I2) who suffered a severe adverse event unrelated to the study after session 7 (we collected data after session 4 on some participants for exploratory analyses)

1b - For I2 CRIS, BBS, L-test and steps/day were not assessed after session 4 so post-treatment data is missing

1c - For I2, no 16-week follow-up data is available due to being withdrawn after session 7

2 - Participant I21 has no post-treatment or follow-up data due to discontinuing intervention after session 3

3a - Participant I18 did not complete baseline WB-PEQ assessment due to error by researcher

3b - Participant I18 did not complete BBS or L-test at post-treatment visit as blinded therapist was unavailable

4 - Participants I8 was unable to be scheduled for return visit to laboratory to assess BBS and L-test

5 - Participant I20 had technical issues with post-treatment activity monitor

6 - Participants C3 and C15 did not receive randomization because we were unable to successfully schedule a return visit to the lab; there is no post-treatment or follow-up data for these participants

7 - Participants C5 and C17 were lost to follow-up (unresponsive to continues communication attempts) during the at home intervention; there is no post-treatment or 16-week follow-up data

8a - Participant C1 did not return to the laboratory to complete post-treatment session due to illness; no post-treatment surveys or activity data is available (had not yet begun mailing surveys and monitors as an alternative)

8b - Participant C1 was contacted by phone to complete the post-treatment CRIS; for CRIS only he is included in baseline vs post-treatment and in post-treatment vs 16 week comparisons

9a- Participant C22 could not be reached by phone for post-treatment CRIS

9b - Participant C22 was lost to follow-up and did not complete any week 16 follow-up data

10a - Participant C16 did not visit lab for post-treatment session; there is no BBS and L-test; did receive and complete post-treatment surveys via mail and CRIS via phone

10b - Participant C16 could not be reached to complete 16-week follow-up for CRIS

11 - Participants I4 and I12 were lost during 16-week follow-up with one exception - participant I12 received and returned activity monitors in the mail (failed to return surveys)

12 - Participant I20 could not be contacted for 16-week follow for CRIS and failed to complete SF36 for 16-week follow-up

**References**

1. Miller WC, Deathe AB, Speechley M. Psychometric properties of the Activities-specific Balance Confidence Scale among individuals with a lower-limb amputation. Arch Phys Med Rehabil. 2003;84(5):656-61.

2. Hafner BJ, Morgan SJ, Askew RL, Salem R. Psychometric evaluation of self-report outcome measures for prosthetic applications. Journal of rehabilitation research and development. 2016;53(6):797.

3. Newell AM, VanSwearingen JM, Hile E, Brach JS. The modified gait efficacy scale: establishing the psychometric properties in older adults. Physical therapy. 2012;92(2):318-28.

4. Weijer R, Hoozemans M, van Dieën J, Pijnappels M. Construct validity and reliability of the modified gait efficacy scale for older adults. Disability and Rehabilitation. 2022;44(11):2464-9.

5. Perera S, VanSwearingen J, Shuman V, Brach JS. Assessing gait efficacy in older adults: An analysis using item response theory. Gait & posture. 2020;77:118-24.

6. Organization WH. International classification of functioning, disability and health: ICF: Geneva: World Health Organization; 2001.

7. Landers MR, Durand C, Powell DS, Dibble LE, Young DL. Development of a scale to assess avoidance behavior due to a fear of falling: the fear of falling avoidance behavior questionnaire. Physical therapy. 2011;91(8):1253-65.

8. Resnik L, Borgia M. Reliability of outcome measures for people with lower-limb amputations: distinguishing true change from statistical error. Physical therapy. 2011;91(4):555-65.

9. Steffen T, Seney M. Test-retest reliability and minimal detectable change on balance and ambulation tests, the 36-item short-form health survey, and the unified Parkinson disease rating scale in people with parkinsonism. Phys Ther. 2008;88(6):733-46.

10. Miller WC, Deathe AB, Harris J. Measurement properties of the Frenchay Activities Index among individuals with a lower limb amputation. Clin Rehabil. 2004;18(4):414-22.

11. Lu W-S, Chen CC, Huang S-L, Hsieh C-L. Smallest real difference of 2 instrumental activities of daily living measures in patients with chronic stroke. Archives of physical medicine and rehabilitation. 2012;93(6):1097-100.

12. Resnik L, Plow M, Jette A. Development of CRIS: measure of community reintegration of injured service members. J Rehabil Res Dev. 2009;46(4):469-80.

13. Resnik L, Borgia M, Ni P, Pirraglia PA, Jette A. Reliability, validity and administrative burden of the community reintegration of injured service members computer adaptive test (CRIS-CAT)”. BMC Medical Research Methodology. 2012;12:1-17.

14. Linda Resnik P, Tian F, Ni P. Computer-adaptive test to measure community reintegration of Veterans. Journal of Rehabilitation Research and Development. 2012;49(4):557.

15. Resnik L, Gray M, Borgia M. Measurement of community reintegration in sample of severely wounded servicemembers. Journal of Rehabilitation Research & Development. 2011;48(2).

16. Coleman KL, Smith DG. Step activity monitor: long-term, continuous recording of ambulatory function. Journal of Rehabilitation Research & Development. 1999;36(1).

17. Arch ES, Sions JM, Horne J, Bodt BA. Step count accuracy of StepWatch and FitBit One™ among individuals with a unilateral transtibial amputation. Prosthetics and orthotics international. 2018;42(5):518-26.

18. Gardner AW, Montgomery PS, Wang M, Shen B. Minimal clinically important differences in daily physical activity outcomes following supervised and home-based exercise in peripheral artery disease. Vascular Medicine. 2022;27(2):142-9.

19. Legro MW, Reiber GD, Smith DG, del Aguila M, Larsen J, Boone D. Prosthesis evaluation questionnaire for persons with lower limb amputations: assessing prosthesis-related quality of life. Arch Phys Med Rehabil. 1998;79(8):931-8.

20. Major MJ, Fatone S, Roth EJ. Validity and reliability of the Berg Balance Scale for community-dwelling persons with lower-limb amputation. Archives of physical medicine and rehabilitation. 2013;94(11):2194-202.

21. Godi M, Franchignoni F, Caligari M, Giordano A, Turcato AM, Nardone A. Comparison of reliability, validity, and responsiveness of the mini-BESTest and Berg Balance Scale in patients with balance disorders. Physical therapy. 2013;93(2):158-67.

22. Deathe AB, Miller WC. The L test of functional mobility: measurement properties of a modified version of the timed “up & go” test designed for people with lower-limb amputations. Physical therapy. 2005;85(7):626-35.

23. Rushton PW, Miller WC, Deathe AB. Minimal clinically important difference of the L Test for individuals with lower limb amputation: A pilot study. Prosthetics and orthotics international. 2015;39(6):470-6.
